# Self-Medication Patterns during a Pandemic: A Qualitative Study on Romanian Mothers’ Beliefs toward Self-Treatment of Their Children

**DOI:** 10.3390/healthcare10091602

**Published:** 2022-08-23

**Authors:** Petruța Tarciuc, Doina Anca Pleșca, Alina Duduciuc, Nicoleta Gimiga, Elena Tătăranu, Valeria Herdea, Laura Mihaela Ion, Smaranda Diaconescu

**Affiliations:** 1 Doctoral School, “George Emil Palade’’ University of Medicine, Pharmacy, Science, and Technology of Târgu Mures, 38 Gheorghe Marinescu Str., 540139 Târgu Mures, Romania; 2 Faculty of Medicine, “Carol Davila” University of Medicine and Pharmacy, 21 Dionisie Lupu Str., 020021 Bucharest, Romania; 3 Clinical Department of Pediatrics, “Dr. Victor Gomoiu” Clinical Children’s Hospital, 21 Basarabia Str., 22102 Bucharest, Romania; 4 Faculty of Communication and Public Relations, National University of Political Studies and Public Administration, 012104 Bucharest, Romania; 5 Faculty of Medicine, “Grigore T. Popa” University of Medicine and Pharmacy, 16 Universitatii Str., 700115 Iasi, Romania; 6 Clinical Department of Pediatrics Gastroenterology, “St. Mary” Emergency Children’s Hospital, 62-64 V. Lupu Str., 700309 Iasi, Romania; 7 Clinical Department of Pediatrics, “Sf. Ioan cel Nou” Emergency Hospital, 720224 Suceava, Romania; 8 Faculty of Medicine, “Titu Maiorescu” University of Medicine, 67A Gheorghe Petrascu Str., 031593 Bucharest, Romania

**Keywords:** self-medication, COVID-19, qualitative study, Romania

## Abstract

Self-medication represents a significant healthcare and health policy issue worldwide, both in developed and underdeveloped countries. Currently, the COVID-19 pandemic is considered a relevant context that could subtly trigger self-medication behavior because of limited access to health care services and the threat of infection with the SARS-CoV-2 virus. While the previous research conducted with quantitative methodologies reported a dramatically increased rate of self-medication around the world, qualitative inquiries on the subjective experience with self-medicine remain scarce in medical and related fields of study. For this purpose, a qualitative study with semi-structured interviews was undertaken to better understand how Romanian mothers (n = 18) applied self-treatment with their children by avoiding medical advice during the COVID-19 pandemic. The results showed that the COVID-19 pandemic did not affect the prevalence of self-medicine among the pediatric population as parents achieved a degree of awareness of self-treatment of their children due to the general context of the outbreak of the COVID-19 pandemic.

## 1. Introduction

Worldwide, more than 50% of all medicines are prescribed, dispensed, or sold inappropriately, while 50% of patients fail to take them correctly. One common source of irrational medicine use is inappropriate self-medication, often of prescription-only medicine [1].

Self-medication (SM) is defined as “the taking of drugs, herbs or home remedies on one’s initiative, or on the advice of another person, without consulting a doctor” [2]. Self-medication involves the use of medicinal products by the consumer to treat self-recognized disorders or symptoms, or the intermittent or continued use of a medication prescribed by a physician for chronic or recurring diseases or symptoms. In practice, it also includes the use of the medication of family members, especially where the treatment of children or the elderly is involved [3].

The key phenomena of self-medication has been widely quantified internationally. In the previous research, self-medication has been conceptualized and quantitatively measured by asking people to report how often they resorted to medication by avoiding medical or formally trained medical advice. For example, a high incident rate was reported in Slovenia (94.9%) and Bangladesh (100%). In European countries, the findings vary: in Czech Republic, the self-medication rate was reported to be approximately 31%, while it was 79% in Spain. In African countries the mean rate was below 67%, while in several Asian countries self-medication was also described as higher (79% in India and 78% in Saudi Arabia) [4,5]. In developed countries, studies on self-medication of children revealed a high prevalence among parents of self-treatment of their children: 25.2% in Germany, 96% in France, 62% in China, 69.2% in Italy, 59% in India, and 56.6% in Brazil. In underdeveloped countries, the prevalence rates were 60% in Yemen, 95.75% in Sudan, and 30.1% in Uganda [6].

The pandemic period is an opportunity to engage in an analysis of self-medication. The result of recent studies are mixed, with some data showing a dramatic increase in self-medication and other findings highlighting unchanged patterns of self-treatment of people with acute health problems. A recent systematic review on the prevalence and correlates of self-medication for the prevention and treatment of COVID-19 revealed the prevalence of self-medication with antibiotics is concerning, with anxiety about contracting the COVID-19 infection being the main trigger for self-medication [7].

At the outbreak of the COVID-19 pandemic, in countries with a full lockdown (n = 590, Norway), a partial (‘intelligent’) lockdown (n = 1004, the Netherlands), and no lockdown (n = 500, Sweden), it was revealed that there were no relevant changes in people’s use of self-management strategies in the prevention of acute illness. Even though the number of people who consulted a medical doctor was low, all consultations were with health care providers for the treatment of COVID-19. The authors conclude that the COVID-19 pandemic did not seem to dramatical change behavior related to consultations with health care providers or the use of self-management strategies, such as dietary supplements and self-help techniques. Most of the respondents usually consumed natural remedies, dietary supplements, and/or self-management strategies but not for preventing or treating COVID-19 [8].

A recent study of Google interest trends for self-medication during the COVID-19 pandemic shows an increase in the number of self-medication searches worldwide since the start of the COVID-19 pandemic, which indicates a higher prevalence of self-medication around the world [9]. Makowska M et al. shows that many people engaged in self-medication for the first time during the pandemic [10]. Self-medication has also become common in Peru [11]. The overall prevalence of self-medication increased from 36.2% before the pandemic to 60.4% during the COVID-19 pandemic among healthcare workers [12].

Self-medication is more common in countries where healthcare systems tend to be less effective because of one or more factors: long waiting times in healthcare facilities, difficulty in obtaining physician appointments, insufficient stock of essential medicines, delay in attention, and an insufficient amount of available beds/space in healthcare facilities, [13]. Probably, all of these conditions worsened during the two years of the COVID-19 pandemic.

According to a survey conducted in Romania (N = 800), 62% of people confronted with minor symptoms (cold, flu, headaches, pain, abdominal pain, sleep problems, stress, and fatigue) resort to self-medication by taking pills that are commonly sold without medical prescriptions (food supplements, vitamins, and minerals). Only 10% of those in the same health situation called doctors for advice regarding the intake of drugs. Then, in the second stage of the research (the focus groups), Romanians interviewees declared that they were familiar with medicines according to previous experiences: they took the medication without medical advice and they viewed that it worked. Qualitative analysis also revealed that Romanians usually calls doctors to ask about their children’s health conditions when the symptoms worsened and became persistent. Romanians turn to self-medication due to individual factors (N = 45) (*it wasn’t a serious health problems; I was confronted with this in the past*) and then to perceived doctor–patient barriers (*It takes time to ask to get in touch with the doctor; I need money for the medical consult; I do not trust doctors*) [14]. It seems that Romanians are very confident in doctors and pharmacist since they get health information only from these main sources: medical (N = 65) and pharmacist (N = 61) advice are accessed rather than the Internet (N = 42) or close friends (N = 36).

A meta-analysis of 543 studies published in PubMed, Scopus, CINAHL, and Web of Science databases until May 2018 and conducted with qualitative methodologies highlighted that the experiences of self-medication are determined by personal, social, organizational, and cultural factors, such as the following: the self-medication is considered cost-effective; self-medication follows friends’, relatives’ and pharmacists’ recommendations (affectivity); self-medication is an effect of the lack of information and communication in health care interactions (inefficiency of the healthcare system); and self-medication is related to the self-treatment history of the disease and, consequently, it is easily applied (oversimplification) [15]. This study is relevant for the particularities and factors related to the Romanian self-medication phenomenon. Usually, urban Romanians (N = 1000) are less oriented toward prevention or health monitoring, especially in the absence of a chronic or acute condition. This ‘procrastination’ behavior (*I go to the doctor when I’m really sick and it hasn’t gone away*) is determined by poor medical education, in general, but also by financial or emotional reasons (avoiding the feeling of vulnerability in contact with doctors or the health system, the fear of not receiving a bad/chronic diagnosis or the desire to avoid the interaction with the public health system) [16]. The limited or restricted access to the health system during the pandemic, as well as Romanians’ fear of not contracting COVID-19 from clinics and hospitals, further exacerbated this behavior of avoiding and postponing health monitoring. Thus, almost half of the urban Romanians went to the family doctor less or not at all (43%) or to specialist doctors less or not at all (50%). At the same time, almost a quarter of Romanians (23%) did not have blood tests, and 29% did not resort to investigations, monitoring, or ultrasounds for monitoring or prevention [16].

The aim of our study is to overcome a limit identified in the previous research, that is the use of quantitative methodologies to understand the prevalence of self-medication of individuals. Currently, little is known about how individuals think about self-medication and how they arrive at it. Therefore, a qualitative study with semi-structured interviews was undertaken to better understand how parents (in this case, mothers) self-treat their children by consciously or unconsciously avoiding medical advice during the COVID-19 pandemic. Specifically, the purpose of our study was to better understand if, how, and why parents avoided medical advice (in this case, mothers) and self-treated their children during the COVID-19 pandemic. 

At the same time, our research represents the second stage of an inquiry on patterns and factors related to self-medication among Romanian parents. In the first stage of the research, we conducted a survey to assess how Romanian parents resort to self-medication, specifically their beliefs and perceived risks of the administration of medicine to their children without medical advice, frequency of self-medication, symptoms, and types of medications most used without medical advice [17]. Even though the parents were aware of the risk of administrating drugs without medical advice, self-medication usually appears when the number of the child’s health issues increases during a certain period (i.e., six months) [14]. Based on these survey data, we further issued new research questions for an in-depth understanding of attitudes and behavior related to self-medication, exactly what are the specific contexts that trigger the use of self-medication of children and how the COVID-19 pandemic affected the self-medication of the pediatric population. Consequently, we formulated new research questions from the literature review and previous quantitative study conducted by the first author: 

*RQ. 1. How does mothers deal with COVID-19 measures, in general and with children’ health issues during COVID-19 states of emergency?*

*RQ. 2. What are the mothers’ perceptions and experiences assign to self-medication?*

*RQ. 3. What are the common situations that determine mothers to resort to the self-treatment of their children?*

*RQ. 4. Does the COVID-19 pandemic leads mothers to self-treat their children?*

## 2. Materials and Methods

To meet the purpose of this research, we employed a qualitative approach, namely grounded theory [18]. Qualitative research gives more freedom to respondents to narrate the subjective experience of self-medication; to be more spontaneous; and to develop their ideas, arguments, and connections on the researched topic. Therefore, the semi-structured interviews collect an in-depth perspective on the issue of self-medication, which could enlarge the insights into the self-medication behaviors and attitudes than those obtained previously via quantitative analysis.

### 2.1. Sampling and Recruitment

We project a purposive sample that fits our first intention to study the self-medication behavior of mothers and who meet our established criteria. Thus, 25 women from a General Practitioner (GP) database were asked telephonically to join an interview on health issues during the COVID-19 pandemic. Of the total, 18 accepted to take part in the study, while 7 declined the invitation. The contact details of the participants were taken from the medical database of the GP. When we selected the participants, particular inclusion criteria were considered: women, with at least one child, from an urban environment. The recruitment stopped when more than 18 women confirmed participation in the study. The sample (n = 18) was considered convenient for an exploratory study on how mothers undertook self-treatment of their children.

All of the persons interviewed in this research are female (n = 18), mothers aged between 26 and 44 years old, with at least one child. Most of them had completed higher education (17 out of 18 respondents). At the date of the interview, one respondent had three children, and in another case the respondent was pregnant with a second child. Each of the interviewees had experience raising and caring for children. All of them had been at the medical office of their family doctor at least once. 

Regarding the occupations of the interviewees, the mothers worked in the legal and economic fields, but some interviewees were self-employed. In one case, one mother stated that she was not employed at the time of the interview. In addition, depending on the age of the minors, some of the mothers (respondents 1, 4, 6, 8) were still on parental leave.

The study was approved by the ethics committee of the Romanian Academy of Medical Sciences (no. 2 SNI/27.02.2019). Because of the pandemic, before the interview, each of the responders received an electronic copy of the informed consent that they signed and sent to their GP via email. 

### 2.2. Data Collection

Data were collected from September to December 2021 using telephone semi-structured interviews. The average of the interview was 45 min. Each interview was recorded with the agreement of the respondent. The discussions were subsequently transcribed and anonymized.

To fulfill the requirements of the research method, a draft version of the interview guide was developed. The guided interview was then revised by four experts from the medical and social science fields who probed the questions and advanced recommendations to arrive at the final version of the guide. In building the guided interview, we considered the previous research results as well as the clinical experience of the coordinator of this research. Overall, the guided interview consists of four sections: demographics; introductory questions (icebreakers) about the pandemic and the participant’s life during the pandemic; interim questions on doctor–patient relations, and questions that assessed self-medication attitudes and behaviors in certain situations. At the end of the interview, participants were invited to add anything they wanted to say on the topic.

The specialists’ feedback was integrated to achieve the expected level of validity and fidelity of the data. The experts agreed that the topic of the research was sensitive and could trigger biased responses. Asking people to report their socially undesirable and sensitive behaviors could cause the participants to respond so as to be viewed favorably by the interviewer. Consequently, we used direct and indirect question techniques to deepen the experience of participants in medicating their children without medical advice. Respondents were asked first to evaluate a presumed situation where a person treats their ill child without medical advice. Secondly, a direct question on self-medication was addressed. To increase the accuracy of the responses, we compared how participants responded to direct as well as indirect questions regarding self-medication. Specific qualitative research criteria and correspondent actions (Supplementary Material Table S1) were also performed to achieve the triangulation, validity, and credibility of the data. 

### 2.3. Data Analysis

As the corpus of the transcript were relatively small-scale, no software was used to code and analyze the unstructured data of the transcripts. Before data collection, all the authors were involved in the initial conversation about the topic. The first (P.T.) and the third (A.D.) authors, together with the team leader (S.D.), were involved in the first round of reading the transcripts and the open coding process. At first, the transcripts underwent three rounds of reading. During these three sessions, authors (P.T., A.D. and S.D.) made a list of emerging codes from the transcripts and grouped them into categories. They identified the common patterns of the transcript. Then, the other four authors (V.H., N.G., D.A.P, L.M.I. and E.T.) evaluated the coding and category process and proposed a way to resolve any disagreement between the authors involving the coding process. After the completion of coding and generative categories, two members of the team (P.T. and A.D.) developed a preliminary codebook, with codes, definitions, categories, and corresponding quotations extracted from the transcripts. Any disagreement between the definitions of the codes and categories was resolved by agreement. The research analysis was made by A.D., P.T. and S.D., and it underwent debate and peer evaluation (V.H., N.G., D.A.P, L.M.I. și E.T.) among the authors until they reached a consensus.

Data analyses were performed using open as well as deductive coding. At the first reading of the transcripts, open coding was involved to add in vivo perspectives of the participants, mainly their insights and words on the pandemic situation, doctor–patient relations during a pandemic, and the healing of their children when they encounter a medical problem. Specifically, the following codes were generated: situations (contexts), health problems, reactions to health problems, behaviors, and solutions to the health problems. In the second reading of the data, a deductive coding process was employed based on the literature review and our research questions. As the coding progressed, data were grouped into certain categories and selective themes were selected based on research questions (Table 1). Specifically, in the second round of the data analysis, the identified codes were group into categories according to our research questions and literature review. For example, attitudes were often measured as a three-dimensional concept [19], that is, the affective, cognitive, and behavioral components. Therefore, the category “attitudes toward self-medication” is made up by the following identified codes: cognition, meanings, experiences, emotion and behaviors assigned to self-treatment of the child.

## 3. Results

### 3.1. The General Impact of Isolation and Quarantine, Family Behavior during COVID-19 Pandemic

Respondents describe changes observed in their daily lives with the onset of the pandemic. Only one interviewee claimed that her life was unchanged (patient 7). The main difficulties perceived by respondents were financial, such as the loss of one of the family members’ jobs, and the insecurity of the job:

*There have been problems or there are financial problems because the husband has lost his job. It was a three-month period in which he looked for another job and it was not easy at all. I work in an NGO…that is, throughout this period, the fear of being left without a job has been seen, the fear of being left without an income, there is a great psychological pressure on us*.

(Patient 11)

Participants also spoke about the consequences of the emergency period on their relations with family members, friends, and relatives. Social contact, trips, and meetings with friends were severely reduced or absent during the COVID-19 pandemic. However, when they contracted the SARS-CoV-2 virus, they valued more what they did and had before the quarantine period. In the context of online education, children felt the need for further clarification, and these were also provided by parents. The shared medical decision regarding the state of children’s health and other household patterns emerged in family behaviors during the COVID-19 period.

*We can say that we faced difficulties, but we spend more time together, as a family time. Before the COVID-19 pandemic, my husband wasn’t involved in parenting. I called the doctor; I struggle with all. Now he can see how hard it is to stay at home hour-by-hour and day-by-day with the kids. Now he is involved in parenting: he doesn’t prepare the kid’s meals, but he does the household*.

(Patient 8)

*We started to appreciate more the time spent in nature because when we were free to travel anywhere… I can say I didn’t appreciate so much a ride in the park or… somewhere at… in a plain*.

(Patient 13)

### 3.2. SARS-CoV-2 Infection

Among the respondents, five said they had tested positive but did not need hospitalization; another six participants mentioned that their spouse, child, or a close person had been infected; and eight of them reported that they did not interact with a person infected with the virus. When the news of infection was received, they referred to the ‘fear’, fear of going out and interacting with others, panic at hearing the result of the PCR test, but also fear of the unknown, a feeling expressed by children who were not always able to express symptoms.

*Panic. Very great panic […] at the time we did the test… Panic because we had my aunt at home, who was old and who was very afraid of being sick. It wasn’t easy at all…it took us a day to get back after the shock (patient 2). It was the unknown who I think marked us all, then a little bit of momentary panic, that we all experienced me my husband and child through the interaction with SARS-CoV-2*.

(Patient 3)

*I was scared when my husband got the virus because he has certain health problems. He had, if I recall correctly, two heart attacks, and I was scared. Fortunately, it evolved very, very well. I mean, he did lose his smell and taste but didn’t have any other symptoms to aggravate his health. After all, it passed smoothly, he didn’t have a serious form of the disease and, as far as I read and understood, people who lose their smell and taste have the light form of the disease compared to the people who start suddenly with fever or…but I’m not sure, no…I just read to be informed but I don’t think anybody knows for sure*.

(Patient 16)

### 3.3. Access to the Healthcare System

Participants referred to the lack of assessment of chronic diseases and the conversion of hospitals in COVID-19 support health centers as a life-threatening situation. However, many of the participants had a positive relationship with their family doctors. Most of the interviewees pointed out that they were in contact with their family doctor via mobile phone or WhatsApp. Regardless of the situation, participants in the study did not resort to self-medication because, as they declared, telemedicine allowed them to solve the situation. There were situations where parents attended the doctors’ office and requested health care and vaccine delivery even if there were a COVID-19 state emergency.

*And I called the doctor and went to her office. Matei was crying very badly, and I didn’t know why he was crying. His belly seemed to be hurting, he seemed to be unable to move*.

(Patient 1)

*I was really in July and August, for vaccines…*.

(Patient 8)

*I know my doctor for many years now. I am her patient since I was a student and I allow myself to call or write a message to her even if it’s something mundane, not serious, I mean, I ask for an appointment. I don’t take antibiotics without a prescription just because…if it’s something easy, I ask my mother who used to be a chemist and she can help with, I don’t know, with something simple like a headache, a toothache, something that doesn’t require antibiotics or powerful drugs. I always ask for medical advice when it comes to using drugs.*.

(Patient 8)

Some participants placed themselves at the opposite pole of their relationship with the doctor: They did not interact with the doctor or request health care because they did not face medical problems. *We didn’t interact much lately; I mean if my little one wasn’t sick, I didn’t call or contact my GP*… (Patient 6).

### 3.4. Source of Medical Information

When we ran the interviews, we also surveyed what type of information sources patients consulted in the medical field, if they asked a family doctor/doctor or turned to other sources. In all situations, the advice of the family doctor was considered very important. Some mothers preferred to access websites to read medical leaflets, to obtain information regarding the diversification of children, or to read about the experiences of other mothers.

Two of the interviewees (patients 10 and 11) claim that they do not search Google because they do not trust the posts, while others (patient 6) considered the online health information posted on Facebook parenting groups as useful:

*Of course, until we came to talk to doctors, to clarify, we read on the Internet and terrified us, …, so I do not recommend that, rather, you speak to a specialist. And we are trying to refrain from doing so*.

(Patient 3)

*I: Tell me if you you’re interested in certain health conditions or if your child was diagnosed with a simple virus, from where do you get the medical information? P6: On the Internet mostly (Patient6). I: Do you search on certain websites you use? P6: No, no. Just Google search. I also have two Facebook groups where I used to ask some questions when my child was a baby. I: But the information was useful? P6: Yes, it works most of the time.*

In the case of a medical emergency, friends or relatives working in medical or related fields were considered sources of information: *I also ask my mother who was a pharmacist and can help us* (Patient 8).

### 3.5. Self-Medication of Pediatric Population

#### Parents’ Attitudes toward Pediatric Self-Medication

Three hypothetical situations were presented to the respondents to assess parents’ attitudes toward pediatric self-medication: first, a friend contacts the survey respondent to ask for her advice because her child had a fever; the second situation assumes that a child had a fever of 39 °C and his mother administered to him an antipyretic (paracetamol/ibuprofen) according to the leaflet; and in the last situation the child had fever of 39 °C and the mother administered an antibiotic (amoxicillin-clavulanic acid). We have used the technique of non-directive questions to get honest answers from the subjects and, above all, for methodological reasons, to avoid the respondents’ bias. When the participants assessed the first situation, the most often advice was to contact the family doctor, but interviewees also advised their friends to use medication first and to contact their doctor or go to the emergency room if the situation did not change:

*Before the pandemic, I would certainly apply a classic treatment when my child was ill, in the sense that I gave him Paracetamol^®^ (paracetamol) because I know that the Paracetamol^®^ (paracetamol) works. I would have waited a day or two, and if I had seen that nothing happens, certainly I would call my family doctor*.

(Patient 19)

*As I always do, I tell them to ask the pediatrician or the GP. In our case, it happened that the GP is also a pediatrician, but the answer is to ask the pediatrician. As far as I’m concerned, I can give some advice from experience regarding food or clothing, but drugs no, because in my case, I wouldn’t medicate as others did, using their dosage or a certain drug even if the disease seems similar. I always ask the doctor first and then I follow his instructions.*

*I: Have you never interfered with a treatment on your own?*

*P8: No, no, and it happened with our friends. They have a baby girl, one or two months older than our child, and she had the same disease as our little one, with fever, and a little rash, but I also told them to ask their doctor. I mean, I can tell them what we used for treatment, but it is not recommended, every child is different, the fever can be higher or lower, and his general state can be different. I also told them first to ask the doctor and follow his instructions and I explained the course of the disease, that after the fever, he will have a small rash/bump and that we didn’t use anything to treat it, they healed on their own. My friends were out of town, and I shared that information with them to calm them down, but that was all I said, I don’t give any advice on medicating, I’m not trained in this field*.

(Patient 8)

*For example, when the symptoms start very suddenly. For a runny nose, I don’t think that I must go to the doctor. Well, now it’s a special situation, but I don’t think that I must visit my GP for a runny nose. I take a pill of Paracetamol^®^ (paracetamol) and I monitor my condition; I drink hot tea or something like that and if it gets worse and I don’t feel well, I will assume that it’s something different and I stop my treatment*.

(Patient 16)

The next situation that was evaluated by the parents was where the child had a fever of 39 °C and the mother administered them paracetamol/ibuprofen according to the prospectus. The responses received can be classified into two categories. The first category made up of participants who claimed that a temperature of 39 °C was high and required the advice of a doctor and the dose of paracetamol/ibuprofen based on the minor’s weight.

*As far as we know, I don’t think he ever had a high fever, past 39 °C, but I know that you treat low fever, 37 °C, 38 °C, with Ibuprofen^®^(ibuprofen), but if he has a high fever, 39 °C and past, he should be seen by a doctor because high fever can be a symptom for a serious disease that needs antibiotics and using Ibuprofen^®^(ibuprofen) will just attenuate the symptom but not treat it. So, yes, I think that for a fever of 39 °C, you should at least ask the doctor and confirm the high fever. For a fever of 37 °C, I have Ibuprofen^®^(ibuprofen) to my little one when he was teething and had passed 36 °C, 37 °C, and 37 °C +, but after I asked the doctor and was told that if he continues to have a fever, when he’s teething, to give him a dosage of 2.5 mL or 5 mL, according to age. He had his first tooth when he has 6 months old, so I gave him a dosage of 2.5 mL. But for high fever, hoe you describe, 39 °C, no, I wouldn’t treat him with Ibuprofen^®^(ibuprofen) so at least I wouldn’t treat after my own opinion*.

(Patient 8)

*39 °C fever for a child is high. The first step is to lower his fever and for that, you need to use an antipyretic, but you also must ask for medical advice. So yes, I’m thinking that fever of 39 °C for a child is a high fever*.

(Patient 16)

*Before a pandemic, I would use the classical treatment, the one I know my child reacts to, so I would give him Paracetamol^®^ (paracetamol) because I know it has the effect I expect, I would monitor his condition for a day or two and if nothing happens, for sure I would contact my GP, as a first step. After a consult, if the doctor says that is something more serious than she expected, she recommends pulmonology or other specialties, if the case requires. My baby girl is predisposed to bronchiolitis and when she’s sick, it manifests very suddenly, or it used to, lately, we didn’t have any problems of this sort, so I would start with the classical cold treatment, which we give to a ten-year-old child,Paracetamol^®^ (paracetamol) a hot bath*.

(Patient 19)

*The child is under the weight that they give in the leaflet and know that doses are calculated, and then I think it is better to call the doctor and give you advice, to give you precise advice, than to experiment on the child*.

(Patient 7)

The second category includes people who agree with the administration of the paracetamol/ibuprofen, and they declare that they applied self-medication because they experienced a similar situation with their first child and the self-treatment worked. These responses show that mothers sometimes give up medical advice and resort to oversimplification based on their own previous experience in healing their children.

Responses varied on their experience in applying self-treatment. Mothers that had two or more children of 3–4 years old did not recommend the administration of paracetamol/ibuprofen at the first signs of disease. The other respondents claimed that they gave this advice from their own experience, which means that self-medication is still recurrent in cases of a medical emergency. In one case, a mother mentioned the possibility of using alternative medical methods to decrease fever, namely, a wet cloth wrapping, which according to her experience, works in decreasing a fever.

*I would recommend them to speak to their family doctor, and if it is not possible or I know… depending on what the value is the fever, give Paracetamol^®^/Ibuprofen^®^/(paracetamol/ibuprofen)that’s what I do*.

(Patient 9)

*I would give him treatment for the fever, and if continued after a day, I would call my family doctor*.

(Patient 16)

When evaluating the last situation, where a child has a 39 °C fever and the mother administers amoxicillin-clavulanic acid, the responses indicate that all mothers are aware that amoxicillin-clavulanic acid, which is an antibiotic and can be administered with physician’s instructions. None of them would recommend or have applied such medication.

*I would not administer antibiotic because I know that antibiotic is administered only in certain cases, for certain infections, and I do not agree to administer this to the child without a prescription*.

(Patient 3)

*I don’t use antibiotics without knowing exactly what it is*.

(Patient 6)

*I would certainly not give the child antibiotics, without knowing what it is all about and without a prescription from the doctor, that is, telling me exactly: Administer the antibiotic x in dose d at I don’t know how many hours, that is, I wouldn’t risk, at least at the first child, no*.

(Patient 8)

*I believe so, yes. I think I have experienced a few times the situation where I needed an antibiotic fast, the one that I knew I react to or my child does, and because I didn’t have it at home, I asked a friend for help, a friend who works in a pharmacy or other medical field. And now, for example, with azithromycin, I didn’t find it the first day I needed it, and the next day, when I had the prescription, I took it from the pharmacy I usually buy my pills, near my building. The family pharmacy to say so, where, in time, you create certain connections/bonds*.

(Patient 19)

## 4. Discussion

The purpose of this study was to explore if, how, and why Romanian mothers resorted to self-treatment of their children during the COVID-19 pandemic. Similar to previous studies [8,10,20], our findings showed that, overall, the COVID-19 pandemic did not lead to a significative change in the ways mothers try to deal with their children’s acute illnesses. It seems that parents achieved a degree of awareness of self-treatment of their children due to the general context of the outbreak of the COVID-19 pandemic. 

Mothers resorted to self-treatment of their children and refused to access health services when they perceived that the health problem of their children was mild or when they had a contact at the pharmacy. As past study [21,22,23,24,25,26,27] also highlighted, mothers often practice self-medication when they consider a disease to be easy or if the episode is similar to a previous illness. Otherwise, contrary to the previous studies [21,22,23,28,29,30], this study reveals the absence of antibiotic self-medication. This could be explained by the Romanian legislation currently in force which ban the sales of antibiotics without a medical prescription. Other drivers of avoiding self-medication with over-the counter-medicine could be in line with the Romanian public health communication campaigns, which raised awareness on the use of unprescribed self-treatment and overuse of antibiotics [27]. 

Mothers use antipyretic or traditional methods, and they use it as a routine in case of flu. Depending on the fever value, they may prefer immediate medical assessment, delayed delivery of the medicine, or administration for two days with subsequent evaluation in case of persistence. This pattern is also described by Fallah Tafti B et al. [31] and Druică et al. [27]. In another study, mothers believe that some symptoms, such as coughing or diarrhea, are symptoms of a minor disease, while others, including fever, indicate a severe disease [22].

Based on the findings of the present study, many of the participants had a positive relationship with their family doctors. The importance of the relationship with the doctor is also encountered in other studies [27,32,33,34,35]. Adequate communication, behavior, and attitudes of health providers can lead parents to use only professional medical care, preventing self-medication [25,34]. Other studies emphasize the role of both the doctor and online sources. Sources of information on social media or other websites are present in the individual’s life and have a certain share in their health according to the context [36,37]. The search for information on the Internet followed by self-medication was confirmed by Fereidouni, Z., et al. [15]. Pharmacists and medicine leaflets are the information sources preferred by other parents [21,31].

In our study, some participants reported that access to health services via telemedicine allowed them to solve the situation and to gain more health literacy on how and when to use medicines. Reduced access to care, surgeries, and other hospital services, combined with fear of exposure to the virus, led to a significant drop in health access. Thus, many diseases that develop symptoms have been managed by using telemedicine. Telemedicine has surged as a feasible tool to maintain patient care; to reduce the risk of COVID-19 exposure to patients, healthcare workers, and the public; to improve health outcomes; and to reduce emergency department visits, including fewer hospitalizations [38,39,40].

Evidence suggests that information about coronavirus and lockdown, fear of infection in health centres and poor accessibility to them, emergency illness, delays in receiving hospital services, distance to the health facility, and proximity of the pharmacy are the reasons for adoption of self-medication [9,41,42,43]. On the contrary, our findings showed that respondents did not delay vaccination, and they went to the medical office for medical assessments. 

Nevertheless, a limit of the current research consists in the homogeneity of respondents and their socio-demographic traits: higher-educated mothers from urban areas. However, how this type of urban population uses self-medication and resorts to self-treatment of their children remains an issue of interest for medical and other related fields of study. Further, a qualitative study could also interview male populations, single-parent families, or chronic patients in order to observe other vulnerable social groups and their patterns related to self-medication.

## 5. Conclusions

Based on our findings and discussion, we derive the main conclusions of the qualitative study.

Firstly, regarding the occurrence of self-medication in the pediatric population during the COVID-19 pandemic, parents managed to avoid self-medication due to medical authorities’ guidelines during the pandemic, which led parents to consult with GP in case of specific symptoms. In the meantime, the threat of SARS-CoV-2 infection enhanced medical services, especially those of the GPs. Consequently, with the overseeing of the GPs, parents cope with their children’s common health problems and prevent self-medication.

Secondly, we observed a high degree of awareness of the self-administration of antibiotics probably due to the current legal regulations in Romania. However, self-medication occurs when a pharmacist facilitates self-medication by ignoring their duties and selling a product in the absence of the client’s medical prescription.

Thirdly, during the COVID-19 pandemic, doctor–patient interaction mediated by telephone and telemedicine eased the uptake of parents’ right therapeutical conduct in case of their children’s health problems and helped them to avoid self-treatment of their children. In light of our data, we can say that structural factors also sustain the correct use of medicine among the public during the COVID-19 pandemic, especially in Central and Eastern Europe, where the role of family medicine could be increased in the general health system [35]. The implementation of the new guidelines for interacting with doctors in case of SARS-CoV-2 infection and the use of telemedicine were good examples of strengthening the role of medical sources in adopting different health behaviors. Otherwise, we cannot say that social media directly affects the administration of drugs without prescriptions. The sources from whom parents seek health information, particularly social media platforms or other websites, have a certain weight in their health decisions, depending on the context.

Fourthly, the presence of a fever is the most common symptom that triggers mothers to apply a specific self-medicine, that is, the administration of antipyretics to their children without medical advice.

Overall, the findings of our research indicate that the COVID-19 pandemic did not affect the prevalence of self-medicine among the pediatric population and parents achieved a certain degree of awareness of applying self-treatment to their children because they become more health-conscious in the general context of the outbreak of the pandemic.

## 6. Patents

This section is not mandatory but may be added if there are patents resulting from the work reported in this manuscript.

## Figures and Tables

**Table 1:** Coding process.

Themes	Category	Free Codes
I. Factors of self-medication associated with the pandemic	1. The general impact of isolation and quarantine	- Limited access to medical care - Economic and financial difficulties - Changes in legislation and restrictions imposed by the authorities
-	2. SARS-CoV-2 Infection	- fear of the disease - family members tested positive - the death of relatives
-	3. Family behaviors	- Time spent with the children - Surveillance of the child by the parents - Shared medical decisions
	4. Access to the healthcare system	- Contact with a family doctor via mobile phone/WhatsApp - Usage of telemedicine - Medical assessment in case of emergency - Reorganization of the medical system (COVID-19 support hospitals)
	5. Source of medical information	- Contact with a medical doctor - Contact with a nurse - Contact with a pharmacist - Information obtained from other parents - Information obtained via the Internet
II. Self-medication of pediatric population	- Attitudes of the parents toward pediatric self-medication	- Subjective meaning of self-medication - Self-medication in a specific context - Past experience with children’s health problems

## Data Availability

Not applicable here.

## Supplementary Materials

The following supporting information can be downloaded at: https://www.mdpi.com/article/10.3390/healthcare10091602/s1, Table S1: Enhancing the quality and the credibility of qualitative analysis.
