# Supplementary material for: Self-Medication Patterns during a Pandemic: A Qualitative Study on Romanian Mothers’ Beliefs toward Self-Treatment of Their Children
Source: Healthcare (Basel). 2022 Aug 23;10(9):1602. doi: 10.3390/healthcare10091602 (PMC9498749; doi:10.3390/healthcare10091602)
Supplement: Supplementary file 1 [file healthcare-10-01602-s001.zip › healthcare-1799239-supplementary.pdf]

Supplementary Material

Table S1. Enhancing the quality and the credibility of qualitative analysis.

	Criteria	Actions
1.	Triangulation	- Using the results of the first stage of the current research to construct new research questions for the second stage of the research (a qualitative inquiry)<br>- The data on self-medication was obtain using multiple source (literature review, different samples for the second phase of the research) and different methods (qualitative and quantitative), such as questionnaire and interviews
2.	Credibility	- The interviews were audio recorded<br>- The guide interview was revised with medical peers and social science experts<br>- The coding process was detailed in the Table 1<br>- The research was conducted with the approval of Ethical Committee of the Romanian Science Academy<br>- The principal investigator has interest of the self-medication topic, i.e. Tarciuc et al. (2020). *Patterns and Factors Associated with Self-Medication among the Pediatric Population in Romania https://doi.org/10.3390/medicina56060312*
3.	Validity	- self-medication phenomenon was addressed via theoretical constructs coined in the past research and limits of the previous research have been identified<br>- filed experts from Romanian Academy approve the study<br>- qualitative analysis was perform taking into account the ”rational subjectivity” of the participants;<br>- the multidisciplinary background of the co-authors allow us to import relevant theoretical constructs on the phenomenon analyzed.<br>- The results of the study were cross-reference and discussed.<br>- Subjective meaning of self-medication behavior as described by the participants were taken into account and cited in the results section<br>- Different interviews techniques (evaluation of other behaviors) were employed to avoid respondents’ cognitive bias
4.	Fidelity	- The limit of the current research was mentioned.<br>- When selecting and recruiting process particular criteria were identify and applied.<br>- The similarities and contrasts with the previous self-medication research findings were identified.
